# Prevalence of Enteroparasites Among Non-Saudis in Bahrah, Saudi Arabia

**DOI:** 10.7759/cureus.9253

**Published:** 2020-07-18

**Authors:** Majed H Wakid

**Affiliations:** 1 Medical Laboratory Technology, Faculty of Applied Medical Sciences, King Abdulaziz University, Jeddah, SAU; 2 Special Infectious Agents Unit, King Fahd Medical Research Center, Jeddah, SAU

**Keywords:** intestinal, parasites, bahrah, saudi arabia, prevalence, risk factors

## Abstract

Background

Enteroparasitic infections in tropical and subtropical regions of the world are among the most common diseases. The majority of the cases may show no symptoms; however, many untreated cases may experience severe complications. The recent substantial economic development in Saudi Arabia has resulted in an inflow of millions of workers with intestinal parasitic infection, from highly endemic countries, mainly from Asia and Africa.

Objective

This cross-sectional study aimed to assess for the first time the prevalence and associated factors of intestinal parasitic infestation among non-Saudi residents in Bahrah, western region, Saudi Arabia.

Materials and methods

A total of 355 stool samples were collected from participants included in this study for several examinations of intestinal parasites. In addition, questionnaires including personal data, sociodemographic data, personal hygiene, health habits and other factors were used.

Results

The prevalence of intestinal parasitic infections among non-Saudi workers in the present study was 22.3%, and the prevalence of protozoan infection was higher than helminth infection. Most of the workers were from Asian countries and were prevalent with Trichuris trichiura, Blastocystis hominis, Endolimax nana and Ascaris lumbricoides. Single intestinal infections were among 86% of the infected cases. The main significant factors associated with intestinal parasitic infections were personal hygiene practices (such as proper handwashing before meals and after using the toilet), source of drinking water and type of living accommodation.

Conclusion

Intestinal parasites were slightly more prevalent among non-Saudi workers in Bahrah. There is a need for public health awareness programs to prevent spreading of the infections.

## Introduction

Infections with intestinal parasites are among the common neglected diseases in many tropical and subtropical regions of the world [1]. These organisms can live, replicate or cause clinical manifestations in the human gastrointestinal system. Globally, the World Health Organization estimated that 24% of the world’s population is infected with soil-transmitted helminth, while more than three billion have no symptoms and over 800 million children are at risk of infection. The intestinal parasitic infection can lead to many severe complications such as malnutrition, growth retardation, several types of anemia, cancer, poor school performance and other problems [2,3]. Most of the intestinal parasites are transmitted mainly among communities with low socioeconomic condition due to poor sanitation and hygiene practices, contaminated food, water and soil. Saudi Arabia's climate is hot most of the year. In addition, many workers in Bahrah are non-Saudis from Asian and African countries, where tropical diseases including enteroparasitic infections are prevalent.

Bahrah is a town in Makkah Province, in the western region of Saudi Arabia, and is located between Makkah and Jeddah. Its population in 2017 according to the General Authority for Statistics was 96,646, of which 71% were Saudis and 29% were non-Saudis (25% males and 4% females) [4].

The present study was conducted to determine for the first time the prevalence and risk factors of intestinal parasites among non-Saudi people living in Bahrah, Saudi Arabia.

## Materials and methods

Study design and sample collection

This cross-sectional study was conducted for a six-month period (March to August 2019) in Bahrah town of the western region of Saudi Arabia. A total of 500 participants were provided with a labeled, clean plastic stool sample collector and instructions for collection. Each one was provided with a consent form and a questionnaire related to personal data, sociodemographic data, health and awareness about intestinal parasites. All samples related to incomplete forms or with insufficient quantity were excluded.

Parasitological analysis of the samples

Sample analysis included gross examination to check for color, consistency, presence of macroscopic blood, mucous, adult worms (of *Ascaris lumbricoides* and *Enterobius vermicularis*) or gravid segments of *Taenia* species.

In addition, stool samples were prepared and examined by different techniques including the direct smears, sedimentation method, trichrome staining and modified Kinyoun’s staining. All wet smears were examined using 10x and 40x objective lenses, while permanently stained smears were examined using the oil immersion objective lens.

Direct smears were prepared by emulsifying 2 mg of stool with saline and iodine on a glass microscope slide [5,6]. Sedimentation technique was performed as described in previous studies with slight modification: 2-3 g of stool sample was emulsified in 10 ml of 10% formal-saline and left for 30 minutes [5,7]. Preserved stool was then passed through two layers of gauze, centrifuged at 2000 rpm for 10 minutes and the supernatant was disposed of. In case of unclear supernatant, the washing step was repeated. The sediment was resuspended in 10 ml formal saline (10% v/v), 3 ml of diethyl ether was dispensed and shaken vigorously for 20 seconds and then centrifuged at 2000 rpm for 10 minutes. The sediment was mixed with two drops of iodine and then examined under a light microscope. Para-Pak® (Meridian Bioscience, Germany) trichrome stain was used and performed based on the manufacturer's procedure [8]. For microscopic detection of *Cryptosporidium*, 5 mg of smeared stool was left to air dry, fixed in alcohol and then stained with carbol fuchsin for 10 minutes. After that, smears were rinsed with tap water, decolorized in 1% sulfuric acid for 1 minute, washed with tap water and counter-stained with methylene blue for 5 minutes. Finally, stained smears were rinsed with tap water, air dried and then examined [6].

Rapid test for *Cryptosporidium* and *Giardia*

Rapid chromatographic immunological detection of *Cryptosporidium* and *Giardia* using the CerTest Biotec (Zaragoza, Spain) rapid card was carried out using stool samples according to the manufacturer's instructions [9].

Fecal occult blood

Fecal occult blood (FOB) using Hemosure® (WHPM Inc., Irwindale, CA) was investigated according to the manufacturer's procedure [10].

Statistical analysis

Statistical analysis was performed using Statistical Package for the Social Sciences (SPSS), version 20 (IBM Corp., Armonk, NY). Demographic data and categorical variables were analysed by descriptive analysis. The chi-square (χ^2^) test was used to analyse the level of significant association between sociodemographic variables and intestinal parasitic infection. A P value < .05 was considered statistically significant.

## Results

Of the 500 participants, the number of overall samples that fulfilled the requirements was 355, while the total number of infected cases with intestinal parasites was 22.3% (79/355) from 10 nationalities with no significant difference (P > .05), as shown in Table 1.

**Table 1:** Nationalities of infected cases X^2^ = 10.76, P > .05

Nationality	Total	Positive cases		
	N (%)	n	% (N=355)	% (n=79)
Bangladeshi	73 (20.6)	21	5.9	26.6
Indian	61 (17.2)	18	5.1	22.8
Pakistani	48 (11.0)	7	2.0	8.9
Yemeni	39 (13.5)	8	2.3	10.1
Egyptian	35 (4.8)	5	1.4	6.3
Sudanese	27 (6.5)	3	0.8	3.8
Indonesian	23 (9.9)	5	1.4	6.3
Ethiopian	18 (5.1)	4	1.1	5.1
Filipino	17 (7.6)	6	1.7	7.6
Nepal	14 (3.9)	2	0.6	2.5
Total	355 (100)	79	22.3	100

As illustrated in Table 2, most participants were males (93%, 330/355), while the remaining 7% (25/355) were females; however, the prevalence rate was higher in females (7/25, 28%) than males (72/330, 21.8%), but without a significant difference (P > .05).

**Table 2:** Gender and infection X^2^ = 0.513, P > .05

Gender	Positive	Negative	Total
Male	72	258	330
Female	7	18	25
Total	79	276	355

The results revealed that the majority (67.1%) were aged 20-39 years, representing about 80% of the infected cases with a significance difference (P < .05) as shown in Table 3.

**Table 3:** Distribution of infection according to age group X^2^ = 16.9, P < .05

Age group (years)	Positive	Negative	Total
	n (%)	n (%)	N (%)
20-29	45 (57.0)	93 (33.7)	138 (38.9)
30-39	19 (24.1)	81 (29.3)	100 (28.2)
40-49	12 (15.2)	59 (21.4)	71 (20.0)
50-60	3 (3.8)	43 (15.6)	46 (13.0)
Total	79 (100)	276 (100)	355 (100)

As shown in Table 4, out of the 79 infected cases, 68 (86.1%) were infected with one parasite including protozoa (41.8%) and helminths (44.3%), while the remaining 13.9% were infected with mixed infections. The common prevalent intestinal protozoan parasite (including mixed infections) was *Blastocystis hominis* (16, 20.3%), followed by *Endolimax nana* (12, 15.2%), both *Entamoeba coli *and *Giardia lamblia* at 7.6% (6), and *Entamoeba​​*​​​​​* histolytica* (3, 3.8%). Using both the permanent staining and the rapid diagnostic test, none of the fecal samples reacted positively for Cryptosporidium parasite. Seven different helminth parasites were detected in this study, of which five were nematodes. The most common helminth parasite, causing mixed infection, was *Trichuris trichiura* (23, 29.1%), followed by *A. lumbricoides* (8, 10.1%) and hookworm (5, 6.3%). There were two positive cases of *Strongyloides stercoralis*, *Schistosoma mansoni* and *Hymenolepis nana*. *Enterobius​*​​​​​​ *vermicularis* adult worms were detected during physical examination in only one sample. There were significant differences in prevalence only among *E. nana* and *T. trichiura* (P < .05).

**Table 4:** Prevalence of intestinal parasites based on nationalities

Parasites	Bangladeshi (n=73)	Indian (n=61)	Pakistani (n=48)	Yemeni (n=39)	Egyptian (n=35)	Sudanese (n=27)	Indonesian (n=23)	Ethiopian (n=18)	Filipino (n=17)	Nepali (n=14)
Single protozoa infection (33)										
Blastocystis hominis (10)	2	1	1	2	1	0	1	1	1	0
Endolimax nana (10)	5	2	1	1	0	0	0	1	0	0
Entamoeba coli (6)	0	1	1	1	1	1	0	0	1	0
Giardia lamblia (4)	1	1	1	0	0	0	1	0	0	0
Entamoeba histolytica (3)	0	1	0	1	1	0	0	0	0	0
Single helminth infection (35)										
Trichuris trichiura (21)	7	7	2	1	0	1	1	1	1	0
Ascaris lumbricoides (8)	1	2	1	1	1	0	1	1	0	0
Hookworm (2)	1	0	0	0	0	0	1	0	0	0
Strongyloides stercoralis (2)	0	1	0	0	0	0	0	0	1	0
Enterobius vermicularis (1)	1	0	0	0	0	0	0	0	0	0
Mixed infections (11)										
B. hominis + Chilomastix mesnili (3)	1	0	0	0	0	0	0	0	1	1
B. hominis + hookworm (3)	1	2	0	0	0	0	0	0	0	0
E. nana + Hymenolepis nana (2)	1	0	0	1	0	0	0	0	0	0
Iodamoeba bütschlii + T. trichiura (2)	0	0	0	0	0	0	0	0	1	1
G. lamblia + Schistosoma mansoni (2)	0	0	0	0	1	1	0	0	0	0
Total (79)	21	18	7	8	5	3	5	4	6	2

Tables 1, 4 illustrate that the most frequent intestinal parasitic infections were reported in Bangladeshis (26.6%) and Indians (22.8%), while the least were among the Nepalis (2.5%). There was no significant difference in relation to nationality and overall infection among workers (P > .05).

The highest prevalence of intestinal parasites was detected in food handlers, farmers and house guards (22.8%, 15.2% and 12.7%, respectively), while the lowest was among the house painters (1.2%), as seen in Table 5. There was no significant difference between intestinal parasitic infection and occupation (P > .05).

**Table 5:** Occupation and infection distribution X^2 ^= 11.10, P > .05

Occupation	Positive	Negative	Total
	n (%)	n (%)	N (%)
Food handler	18 (22.8)	46 (16.7)	64 (18.0)
Farmer	12 (15.2)	41 (14.9)	53 (14.9)
House guard	10 (12.7)	33 (12.0)	43 (12.1)
Driver	10 (12.7)	26 (9.4)	36 (10.1)
Electrician	4 (5.1)	28 (10.1)	32 (9.0)
Puncture repair	2 (2.5)	23 (8.3)	25 (7.0)
Cleaner	4 (5.1)	17 (6.2)	21 (5.9)
Barber	2 (2.5)	19 (6.9)	21 (5.9)
Car mechanic	4 (5.1)	14 (5.1)	18 (5.1)
House maid	5 (6.3)	9 (3.3)	14 (3.9)
Housewife	2 (2.5)	9 (3.3)	11 (3.1)
House painter	1 (1.3)	7 (2.5)	8 (2.3)
Carpenter	2 (2.5)	3 (1.1)	5 (1.4)
Plumber	3 (3.8)	1 (0.4)	4 (1.1)
Total	79 (100)	276 (100)	355 (100)

For personal hygiene practices, 71.8% of the participants used to wash their hands before meals either with soap (113, 31.8%) or without soap (142, 40%), and 53 (14.9%) rarely washed their hands, while the rest 47 (13.2%) had the bad habit of not washing their hands. Almost 85% had a conduct of handwashing after using toilet (including 58% without soap). Nevertheless, none of these findings reached a significance level at P > .05. Similar results were found for the habit of fingernail trimming and close contact with animals, as shown in Table 6.

**Table 6:** Personal hygiene practices and risk of infection *X^2^ = 10.7, **X^2 ^= 29.6, ***X^2^ = 19.9, ****X^2^ = 8.7, *****X^2^ = 8.16, P < .05

Practice	Positive	Negative	Total
	n (%)	n (%)	N (%)
Washing hands before meals*			
Yes, with soap	24 (30.4)	89 (32.2)	113 (31.8)
Yes, without soap	23 (29.1)	119 (43.1)	142 (40.0)
Rarely	20 (25.3)	33 (12.0)	53 (14.9)
No	12 (15.2)	35 (12.7)	47 (13.2)
Washing hands after using toilet**			
Yes, with soap	15 (19.0)	77 (27.9)	92 (25.9)
Yes, without soap	36 (45.6)	171 (62.0)	207 (58.3)
Rarely	28 (35.4)	28 (10.1)	56 (15.8)
Washing fresh vegetables/fruits***			
Yes	19 (24.1)	124 (44.9)	143 (40.3)
Rarely	42 (53.2)	131 (47.5)	173 (48.7)
No	18 (22.8)	21 (7.6)	39 (11.0)
Fingernail trimming****			
Always	12 (15.2)	87 (31.5)	99 (27.9)
Sometimes	50 (63.3)	149 (54.0)	199 (56.1)
Rarely	17 (21.5)	40 (14.5)	57 (16.1)
Animal contact*****			
Always	31 (39.2)	156 (56.5)	187 (52.7)
Sometimes	24 (30.4)	53 (19.2)	77 (21.7)
Rarely	13 (16.5)	40 (14.5)	53 (14.9)
Never	11 (13.9)	27 (9.8)	38 (10.7)
Total of each	79 (100)	276 (100)	355 (100)

In the present study, it was found that the source of drinking water and eating at home were statistically significant factors in intestinal parasitic infections (P < .05), as shown in Table 7.

**Table 7:** Source of drinking water, food and risk of infection *X^2^ = 18.6, **X^2^ = 43.8, P < .05

Factor	Positive	Negative	Total
	n (%)	n (%)	N (%)
Regular source of drinking water*			
Bottled	10 (12.7)	41 (14.9)	51 (14.4)
Filtered	11 (13.9)	55 (19.9)	66 (18.6)
Tap	48 (60.8)	175 (63.4)	223 (62.8)
Wells	10 (12.7)	5 (1.8)	15 (4.2)
Cooking and eating at home**			
Always	17 (21.5)	174 (63.0)	191 (53.8)
Sometimes	56 (70.9)	88 (31.9)	144 (40.6)
Rarely	5 (6.3)	10 (3.6)	15 (4.2)
Never	1 (1.3)	4 (1.4)	5 (1.4)
Total of each	79 (100)	276 (100)	355 (100)

Data from Tables 8, 9 suggest higher percentages of parasitic infection in participants who are not aware about the parasites, and those who have arrived recently to Saudi Arabia, but without statistical difference (P > .05).

**Table 8:** Awareness about intestinal parasites and infection X^2^ = 4.67, P > .05

	Positive	Negative	Total
Aware	5	27	32
Not aware	31	136	167
Not sure	43	113	156
Total	79	276	355

**Table 9:** Distribution of infection and years of residency in Saudi Arabia X^2^ = 5.58, P > .05

Year(s)	Positive (n)	Negative (n)	Total (n)
<1	27	64	91
1-5	41	147	188
6-10	6	36	42
>10	5	29	34
Total	79	276	355

Also, as see in Table 10, the infection rate among workers who live with their families (12/112, 10.7%) was less than that for those who live with other workers (38/175, 21.7%) or alone (29/68, 42.7%), with statistical difference (P < .05).

**Table 10:** Accommodation type and infection distribution X^2^ = 24.99, P < .05

Accommodation type	Positive	Negative	Total
With other workers	38	137	175
With family	12	100	112
Alone	29	39	68
Total	79	276	355

Out of the 79 positive cases, 45 were positive for FOB, but without significant association (P > .05), as illustrated in Table 11.

**Table 11:** Fecal occult blood and infection X^2^ = 2.653, P > .05

Occult blood	Positive	Negative	Total
Positive	45	163	208
Negative	34	113	147
Total	79	276	355

## Discussion

This is the first study regarding the prevalence of intestinal parasitic infections among non-Saudi workers in Bahrah, Saudi Arabia. The World Health Organization attributed the spreading of intestinal parasites to several factors such as the status of personal hygiene, level of environmental sanitation, drinking water sources, health education practices and control programs [11].

The prevalence of intestinal parasites in this study was 22.3% among 355 workers from seven Asian countries and three African countries. Previous studies conducted in Saudi Arabia reported various prevalence values. Studies in Riyadh, Al-Khobar, Jeddah, Al-Madina and Makkah reported a prevalence of 41.4%, 31.4%, 50.15%, 14.9% and 16%, respectively [12-16]. Like our findings, all these previous studies found that the majority of workers were from Asian countries, within the age range of 20-40 years.

In this study, among the 14 detected parasites, seven were protozoa and seven were helminths, of which, five were nematodes, one cestoda and one trematoda. A total of 68 participants were positive with one intestinal parasite, while 11 were positive for two parasites. This is in agreement with previous studies in Saudi Arabia, where the single infection rate is higher than the mixed infection rate [12-16]. In the present study, none of the cases revealed *Cryptosporidium *parasites. This opportunistic coccidian parasite is common among immunocompetent and immunocompromised individuals and transmission occurs mainly by contaminated drinking water or swimming pools [17,18].

This study revealed that the major detected parasites were *T. trichiura* (29. 1%), *B. hominis* (20.3%), *E. nana* (15.2%) and *A. lumbricoides* (10.1%). Riyadh and Al-Khobar studies reported that *T. trichiura*, hookworm and *A. lumbricoides* were the most common isolated parasites [12,13]. On the other hand, a Jeddah study showed that the most common organisms were *B. hominis*, hookworm and *T. trichiura*, while in Al-Madina were *G. lamblia*, *E. histolytica* and *T. trichiura*​​​​​​, and in Makkah were *B. hominis*, *E. coli* and *G. lamblia* [14-16].

In the present study, at least one participant from each nationality (except Sudanese), was infected with *B. hominis*. Although this parasite is common worldwide, its pathogenicity is still uncertain with great controversy [19-23].

The detected pathogenic protozoa parasites (*E. histolytica* and *G. lamblia*) were detected in 11.4% of the infected cases, while nonpathogenic organisms (*E. nana, E. coli, C. mesnili* and* I. bütschlii*) were detected in more cases (29.1%). Nonpathogenic intestinal protozoa should not be neglected and should considered as a health concern for the infected person, as their oral-fecal route of transmission is similar to the pathogenic organisms. This explains the detection of many mixed infections between pathogenic and nonpathogenic organisms leading to abdominal symptoms [24,25].

This study, consistent with previous studies in Saudi Arabia, revealed that the common detected parasites were protozoa and nematodes. The obvious explanation is the simple and direct mode of infection with these parasites. On the other hand, *S. mansoni* infection in two participants from Africa (Egypt and Sudan) was mainly contracted from their hometowns, as this fluke requires snail intermediate hosts, which are not found in Bahrah, to form the infective cercariae [26,27]. A similar observation was reported in previous studies in Jeddah and Al-Madina [14,15].

Although in this study the majority of workers were from Asian regions, there was not any statistically significant difference between nationality and the intestinal parasitic infection. That was not in agreement with previous studies [15]. A similar finding was observed regarding gender, age and occupation.

Findings from this study revealed that all investigated personal hygiene practices, source of drinking water/meals and type of accommodation showed significant association with intestinal parasitic infection. None of the previous studies on non-Saudi workers investigated these factors.

Results of this study indicated similar previous observations that there was no significant association between intestinal parasitic infection and positive FOB, but were inconsistent with another investigation [28-30].

## Conclusions

This study provides insights about the prevalence of intestinal parasites and clarifies related factors among non-Saudi workers in Bahrah. The prevalence was slightly high (22.3%) and was associated mainly with hygiene practices, individual habits and awareness. Among the positive cases, almost 86% were single infections and 14% mixed infections. Intestinal protozoa and nematodes were the most predominant parasites, followed by cestodes and trematodes.

These findings draw attention for the need of more awareness about diagnosis protocols, control of intestinal parasitic infection, in addition to personal hygiene, eating habits and lifestyle.
